# Supplementary material for: The causal relationship between the human gut microbiota and pyogenic arthritis: a Mendelian randomization study
Source: Front Cell Infect Microbiol. 2024 Nov 26;14:1452480. doi: 10.3389/fcimb.2024.1452480 (PMC11629706; doi:10.3389/fcimb.2024.1452480)
Supplement: Supplementary file 1 [file DataSheet1.zip › mendelian test/ebi-a-GCST90027481.csv_forest.pdf]

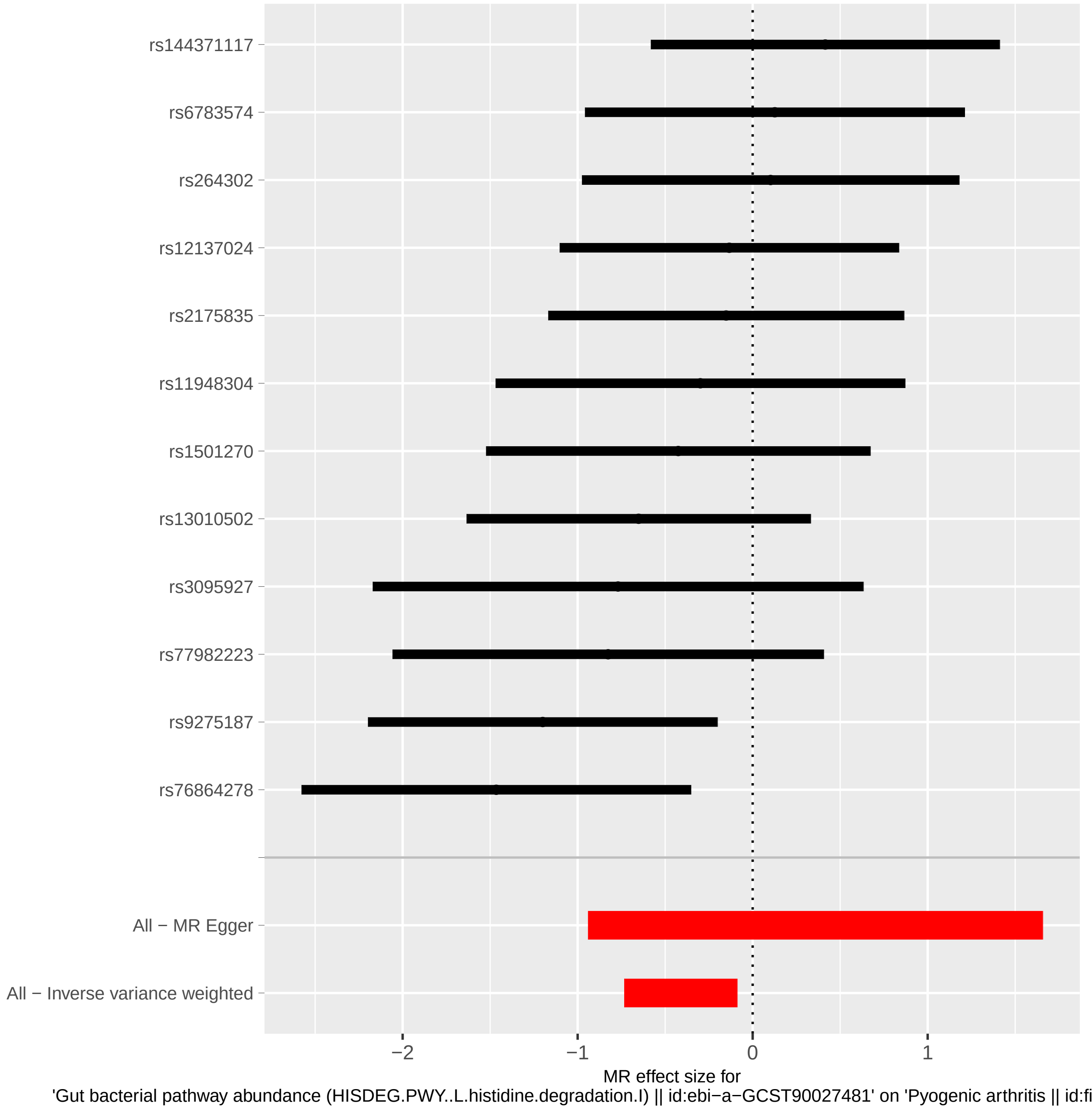

rs144371117
rs6783574
rs264302
rs12137024
rs2175835
rs11948304
rs1501270
rs13010502
rs3095927
rs77982223
rs9275187
rs76864278
All − MR Egger
All − Inverse variance weighted
−2
−1
0
1
MR effect size for
'Gut bacterial pathway abundance (HISDEG.PWY..L.histidine.degradation.I) || id:ebi−a−GCST90027481' on 'Pyogenic arthritis || id:fi
